# Benchmarking Cesarean Section Trends: A Case Study from Tu Du Hospital Using Robson’s Model

**DOI:** 10.3390/healthcare13162070

**Published:** 2025-08-21

**Authors:** Hai Thanh Pham, Thanh Quang Le, Nam Hoang Tran

**Affiliations:** 1 Directorate, Tu Du Hospital, 284 Cong Quynh, Ho Chi Minh City 700000, Vietnam; 2 Research Center for Higher Education, Tokushima University, Tokushima 770-8502, Japan

**Keywords:** cesarean section rate, Robson classification (RTGCS), Tu Du hospital, Vietnam

## Abstract

**Background:** Cesarean section (CS) is a critical surgical procedure in obstetrics but is increasingly overused worldwide. Vietnam has seen rising CS rates, especially in urban tertiary hospitals, with limited standardized analysis to guide interventions. This study assesses CS rates at Tu Du Hospital using the WHO-endorsed Robson 10-Group Classification System. **Methods:** A cross-sectional descriptive study was conducted over one month in 2017 at Tu Du Hospital, a major obstetrics referral center in southern Vietnam. All women who delivered during this period were classified into Robson’s 10 groups based on parity, gestational age, labor onset, presentation, fetal number, and prior CS. CS rates and group-specific contributions were analyzed. **Results**: Among 5287 deliveries, the overall CS rate was 42.6%. Group 5 (previous CS) contributed 29.7% of all CSs, followed by Group 2 (nulliparous, induced/pre-labor CS, 26.2%) and Group 1 (nulliparous, spontaneous labor, 12.8%). Failed induction, fetal distress, and cephalopelvic disproportion were common indications. Only 22% of eligible women in Group 5 were offered a trial of labor after cesarean (TOLAC), although the success rate for vaginal birth after cesarean (VBAC) was 67%, indicating underutilization of this option. **Conclusions**: This study provides rare Robson-based evidence from Vietnam, identifying key target groups for intervention. The findings support expanded use of VBAC and more stringent criteria for induction. Future research should explore behavioral and systemic drivers of high CS rates to guide national policy.

## 1. Introduction

Cesarean section (CS) is one of the most commonly performed surgical procedures worldwide. While it is an essential and often life-saving intervention for managing childbirth complications, the rising global trend in CS rates has raised growing concern among clinicians, policymakers, and public health stakeholders. The World Health Organization (WHO) has long maintained that CS rates higher than 10–15% at the population level are not associated with reductions in maternal and neonatal mortality [1]. However, according to the most recent global estimates, the average CS rate reached 21.1% in 2021 and is projected to rise to 28.5% by 2030, with the highest rates observed in Latin America, the Caribbean (over 40%), and parts of Asia and the Middle East [2].

This increase is attributed to a combination of factors, including delayed childbearing, increased maternal comorbidities, medicolegal pressures, patient preferences, financial incentives in private healthcare settings, and perceived safety concerns regarding vaginal birth. Notably, studies have shown that unnecessary cesarean sections can lead to increased risks of postpartum infection, hemorrhage, uterine rupture in subsequent pregnancies, and long-term complications such as abnormal placental implantation [3,4]. Furthermore, overuse of CS places an economic burden on already strained healthcare systems, particularly in low- and middle-income countries [5].

Vietnam exemplifies many of these global trends, with CS rates rising sharply over the past two decades and presenting unique challenges in both urban and rural healthcare settings. In Vietnam, CS rates have mirrored global patterns but with distinct urban-rural disparities. The national CS rate has increased significantly—from 19.4% in 2002 to 36.3% in 2020, with urban tertiary hospitals often reporting rates exceeding 50% [6]. This trend is more pronounced in public and private institutions located in major cities such as Hanoi, Ho Chi Minh City, and Da Nang. Socioeconomic status, maternal education, and institutional practices have all been identified as contributing factors to the country’s rising CS rates [7,8,9]. For example, a study revealed a CS rate of 57.8% in private hospitals [7]), while urban-rural analyses have shown maternal request, fear of labor pain, and provider convenience as common non-medical drivers of cesarean use [9].

The Robson 10-Group Classification System (RTGCS) has emerged as the global gold standard for monitoring and comparing CS rates. It classifies all deliveries into 10 mutually exclusive and fully inclusive groups based on key obstetric characteristics such as parity, gestational age, fetal presentation, labor onset, and number of fetuses [10,11,12]. By enabling standardized comparisons across different settings and over time, the Robson system helps identify target groups for intervention and offers a pathway toward evidence-based, locally adapted CS reduction strategies.

Although national and regional studies have highlighted the rising trend in CS rates in Vietnam, several gaps remain in the literature:

First, there is limited use of standardized international classification systems, such as the RTGCS, to analyze CS rates across Vietnamese hospitals.

Second, few studies provide granular, group-specific data on obstetric populations within Vietnam, especially in tertiary referral settings where clinical decision-making complexity is highest.

Third, there is a lack of recent institutional audits using robust methodologies to identify drivers of both primary and repeat cesarean deliveries.

These limitations hinder efforts to compare trends across time and between institutions, and to develop targeted, evidence-based interventions to reduce unnecessary CS.

This study was conducted at Tu Du Hospital, the top referral center for maternal care in southern Vietnam. Using Robson’s classification, the study aimed to: (1) determine the cesarean section rate during a representative time period, (2) identify the primary contributing Robson groups, and (3) explore the major clinical indications behind cesarean decisions. The findings are expected to inform not only local quality improvement efforts but also contribute to national and regional dialogue on optimizing cesarean use in Vietnam’s evolving health landscape.

## 2. Materials and Methods

A cross-sectional descriptive study was conducted at Tu Du Hospital, Vietnam, over a one-month period from 1 June to 30 June 2017. Tu Du Hospital is the largest tertiary-level obstetrics and gynecology center in southern Vietnam, managing approximately 60,000 births annually and serving as a national referral center for high-risk pregnancies.

### 2.1. Study Population and Inclusion Criteria

The study included all women who delivered at the hospital during the study period, provided they met the inclusion criteria. Deliveries with incomplete or missing data relevant to Robson classification were excluded from the analysis. Inclusion criteria were:

- Gestational age ≥ 22 weeks, consistent with Vietnam’s legal threshold of fetal viability.
- Newborn birth weight ≥ 500 g, aligning with WHO reporting standards.
- Availability of complete clinical and demographic data in the hospital’s electronic medical record (EMR) system.
- Women were excluded if their delivery records were incomplete, duplicated, or missing critical variables (e.g., parity, presentation, gestational age, or type of labor onset).

### 2.2. Data Collection

Data were retrospectively extracted from Tu Du Hospital’s EMR system by trained medical staff using a structured data abstraction form. All delivery records were sourced from Tu Du Hospital’s EMR system, which includes mandatory structured fields for key obstetric variables such as parity, gestational age, prior cesarean section, labor onset, and delivery mode. Variables collected included:

- Maternal demographics: age, parity, history of previous cesarean sections.
- Pregnancy and delivery characteristics: gestational age, fetal presentation, number of fetuses, mode of labor onset (spontaneous, induced, or pre-labor CS), and final delivery mode (vaginal or cesarean).
- Indications for CS: documented by attending obstetricians, including fetal distress, cephalopelvic disproportion (CPD), failed induction, abnormal fetal lie, breech presentation, and maternal request.

To ensure data reliability, a random 10% sample of records was independently cross-verified by a second reviewer. Discrepancies were resolved by consensus, involving a senior obstetrician when necessary.

### 2.3. Classification Framework

All deliveries were classified according to the RTGCS, which is endorsed by the WHO for monitoring and comparing CS rates across healthcare facilities [12,13]. This system groups women based on six key obstetric characteristics:

- Parity (nulliparous vs. multiparous),
- Onset of labor (spontaneous, induced, or CS before labor),
- Gestational age (term vs. preterm),
- Fetal presentation (cephalic, breech, or transverse),
- Number of fetuses (singleton vs. multiple), and
- History of prior CS.

Each case was assigned to one of ten mutually exclusive groups. Group size, CS rate within the group, and each group’s relative contribution to the overall CS rate were calculated.

### 2.4. Statistical Analysis

Descriptive statistical methods were used to summarize the data. Frequencies and percentages were calculated for categorical variables such as Robson groups and delivery methods. The overall CS rate was calculated as the number of CS deliveries divided by the total number of births during the study period.

To assess group-specific contributions to the overall CS rate, the following metrics were computed:

- Relative size of each Robson group (proportion of total deliveries).
- CS rate within each group (proportion of CS in each group).
- Contribution of each group to total CS (proportion of overall CS attributable to each group).

Data analysis was conducted using IBM SPSS Statistics version 22.0 (IBM Corp., Armonk, NY, USA). Continuous variables (e.g., maternal age) were expressed as means and standard deviations if normally distributed; medians and interquartile ranges were used otherwise.

## 3. Results

### 3.1. Group Distribution and Contributions to Overall CS Rate

During the one-month study period from 1 June to 30 June 2017, a total of 5287 women delivered at Tu Du Hospital. Of these, 2254 underwent CS, resulting in an overall CS rate of 42.6%. All cases were classified into the RTGCS, with no women excluded due to missing classification criteria. Table 1 summarizes the distribution of deliveries and CS rates across the ten Robson groups.

Group 5 (multiparous women with a previous CS, singleton, cephalic, ≥37 weeks) had the highest contribution to overall CS deliveries, accounting for 670 of 2254 CSs (29.7%) and a CS rate of 79.7% within the group. This high rate reflects both clinical caution regarding trial of labor after cesarean (TOLAC) and institutional practice patterns. Among the 841 women in this group, only 186 were offered vaginal birth after cesarean (VBAC), with a success rate of 67%, suggesting underutilization of TOLAC opportunities despite favorable outcomes.

Group 2 (nulliparous, singleton, cephalic, ≥37 weeks, induced or pre-labor CS) was the second-largest contributor, responsible for 591 CSs (26.2%). The CS rate within this group was 86.2%, indicating that most women who were either induced or scheduled for elective CS did not progress to vaginal birth. The high rate suggests potential overuse of elective CS and highlights the need for stringent criteria for labor induction in nulliparous women.

Group 1 (nulliparous, singleton, cephalic, ≥37 weeks, spontaneous labor) contributed 289 CSs (12.8%) out of 1470 women, with a group-specific CS rate of 19.7%. Detailed chart review revealed that 59 out of 96 CSs in this group were due to non-reassuring fetal heart rate patterns, suggesting a potential over-reliance on cardiotocography without adjunct diagnostic tools.

Group 10 (all singleton, cephalic, <36 weeks, including previous CS) contributed 214 CSs (9.5%) with a moderate CS rate of 40% within the group. This reflects the clinical complexity of preterm deliveries, where fetal compromise and failed induction are common reasons for surgical intervention.

Groups 6 and 7 (all breech presentations) showed high CS rates—84.7% and 81.5%, respectively—which aligns with international standards following the decline of routine vaginal breech delivery after the Term Breech Trial. Although these groups only contributed 7.6% to total CSs, their nearly uniform CS approach reflects prevailing clinical conservatism.

Group 3 and Group 4 (multiparous women without previous CS) had the lowest CS rates—4.7% and 46.0%, respectively—and contributed only 7.5% to overall CS cases combined. These findings confirm that spontaneous labor in multiparous women remains the most protective scenario against CS.

Group 8 (all multiple pregnancies) and Group 9 (all abnormal lies) had CS rates of 72.1% and 35.9%, respectively. Although these groups represented a small proportion of the total population (2.3% and 3.2%), their elevated CS rates highlight the inherent obstetric risk associated with these presentations.

### 3.2. Key Trends and Observations

Primary CSs (Groups 1 and 2) contributed to 39% of all cesarean deliveries, underscoring the importance of optimizing labor management in nulliparous women. These groups represent a key target for interventions aimed at reducing first-birth CSs and subsequently lowering rates in Group 5 (repeat CS).

Despite national and institutional interest in promoting VBAC, only 22% of eligible women in Group 5 were offered a trial of labor, indicating a gap between policy and practice.

Failed induction, non-reassuring fetal heart rate, and cephalopelvic disproportion (CPD) were the top three indications for CS across multiple groups. The frequency of these indications—particularly in cases of induced labor and pre-labor CS—raises concerns about subjective diagnosis, timing of intervention, and the adequacy of diagnostic criteria.

The combined CS rate for breech, multiple pregnancy, and abnormal lie groups (Groups 6–9) was 81.2%, demonstrating adherence to contemporary recommendations but also suggesting that facilities with higher technical capacity may consider reintroducing selective vaginal delivery with strict criteria.

## 4. Discussion

The CS rate of 42.6% at Tu Du Hospital, as observed in this study, is substantially higher than the WHO-recommended optimal rate of 10–15% and reflects the broader global trend of rising CS rates, particularly in middle-income and urban settings. Our findings are consistent with recent international analyses that show CS rates exceeding 40% in several middle- and high-income countries, including Brazil, Egypt, Turkey, and China [1,14]. International comparisons in this study primarily reference data from 2015 to 2017 to ensure temporal alignment with our 2017 dataset. In Vietnam, this figure aligns with the urban trend, as recent Ministry of Health data and population surveys report CS rates ranging from 35% to 60% in metropolitan regions, with private sector rates often exceeding those in public hospitals [6,9,15].

A particularly concerning observation from this study is the dominant contribution of Robson Group 5 (women with a previous CS), which accounted for 29.7% of all CSs. This finding echoes patterns in other countries where repeat cesareans constitute the largest share of surgical deliveries. For instance, data from China [16,17] and Iran [18] show that more than 35% of CSs arise from repeat cases. While the WHO and FIGO advocate for trial of labor after cesarean (TOLAC) in appropriately selected women, our data reveal that only 22% of eligible women were offered VBAC at Tu Du Hospital—despite a relatively high success rate of 67%. This low offering rate may reflect a combination of institutional and systemic factors. First, in high-volume tertiary hospitals like Tu Du, there is often a conservative clinical culture that prioritizes predictability and risk minimization, particularly for women with a prior cesarean. Similar patterns have been observed in both high- and middle-income settings. For instance, in a U.S. study, although VBAC was deemed generally safe for many women, physician practices varied significantly due to perceived medico-legal risks and clinical uncertainty [19]. In contrast, in our study, only 22% of eligible women in Group 5 were offered a TOLAC, revealing more restrictive practices compared to international benchmarks. Second, institutional and health system barriers play a critical role. A qualitative study in Iran identified major challenges such as inadequate hospital infrastructure, absence of standard guidelines, and limited staff training—all of which discouraged healthcare providers from offering VBAC [20]. This closely aligns with our local context, where similar systemic gaps persist. Third, provider training and counseling are often inconsistent. In many cases, the lack of standardized, evidence-based protocols for screening VBAC eligibility and managing intrapartum complications contributes to provider hesitancy [21]. Given the complexity of TOLAC, clinicians may prefer the predictability of scheduled cesarean deliveries, especially when continuous fetal monitoring or immediate access to surgical intervention is unavailable. To promote more equitable access to VBAC, health systems must address these barriers through structured clinical pathways, staff training, and improved communication with patients. Creating multidisciplinary teams, ensuring legal protection for evidence-based practice, and integrating patient education could help increase the safe uptake of VBAC in settings similar to ours.

Primary cesarean sections, particularly in nulliparous women (Groups 1 and 2), contributed 39% of all CSs in our study. This mirrors the international concern that reducing first-birth CSs is key to curbing overall CS trends. In countries like Sweden and the Netherlands, where Group 1 CS rates are kept below 10%, robust midwifery care, consistent use of partographs, and strong social support networks have been effective in promoting vaginal births [22]. In contrast, the failed induction rate in Group 2b (86.2%) is notably high compared to international benchmarks and likely reflects institutional factors at the time of data collection. Importantly, standardized readiness assessments such as Bishop score were not consistently documented in the hospital’s records, limiting clinicians’ ability to objectively determine cervical favorability prior to induction. Moreover, there was no unified induction protocol in place; methods varied depending on provider discretion and included agents such as oxytocin and prostaglandin analogues. The lack of standardized assessment and protocol likely contributed to the elevated failure rate. This finding highlights a critical area for clinical quality improvement, namely, the adoption of evidence-based induction guidelines and the routine use of cervical readiness scoring to guide clinical decision-making. The high rate of pre-labor CS or induction failures in Group 2 at our hospital (86.2%) suggests a need to reevaluate induction criteria, cervical readiness assessments, and labor support practices. A Cochrane review found that misoprostol and Foley catheter-based protocols may significantly reduce the risk of failed inductions, particularly in nulliparous women [23].

While cross-country comparisons with systems such as Sweden, the Netherlands, the UK, and Canada offer useful benchmarks, it is important to contextualize these findings within Vietnam’s healthcare landscape. Unlike midwife-led systems in some high-income countries that promote physiologic childbirth and limit intervention, Vietnam’s maternity care remains largely physician-led, particularly in tertiary hospitals like Tu Du [7,24]. Midwives have limited autonomy in clinical decision-making, and shared decision-making models are not yet uniformly implemented. Additionally, while Vietnam has a universal health insurance scheme, reimbursement structures and out-of-pocket costs may influence delivery choices, especially when combined with growing access to private care [25]. These structural differences should be taken into account when applying international models of cesarean reduction to the Vietnamese context.

The high CS rates in Groups 6–9 (breech, multiple pregnancy, abnormal lie) reflect adherence to international clinical guidelines that favor cesarean delivery in complex or mispresented cases. However, emerging studies suggest that with appropriate case selection and provider expertise, selective vaginal breech delivery may be safely reintroduced in high-resource settings [26]. Given Tu Du Hospital’s capacity and referral status, further exploration into individualized delivery planning in these groups may be warranted.

The CS rate for Group 10 (preterm singleton cephalic births) was 40%, which is lower than in comparable facilities in countries like India, China, and Indonesia [27]. This may reflect improved neonatal support at Tu Du Hospital, allowing for more tolerance of labor in early gestations. Nevertheless, careful balancing of risks related to neonatal outcomes versus maternal surgical complications must guide CS decisions in preterm cases.

From a national perspective, Vietnam has witnessed a consistent rise in CS rates over the last two decades, driven by factors such as maternal request, perceived safety, provider convenience, and urbanization [7,28]. A 2021 study found that maternal education and urban residency were among the strongest predictors of elective CS, even in the absence of medical indications. Additionally, cultural beliefs, fear of pain, and commercialization of childbirth—particularly in private hospitals—have contributed to non-medically indicated CSs [28,29]. These trends necessitate both clinical and public health interventions. Standardizing Robson classification auditing across hospitals, investing in antenatal counseling, and developing nationwide CS monitoring dashboards are promising strategies for Vietnam to achieve a more sustainable and equitable model of childbirth.

At the institutional level, Tu Du Hospital may benefit from implementing a multidisciplinary CS reduction taskforce involving obstetricians, midwives, anesthetists, and perinatal educators. Such models have demonstrated success in the UK and Canada, where team-based reviews of CS indications, second-opinion policies, and training in labor support techniques led to significant CS reductions [30,31]. Moreover, leveraging health information systems to automate Robson group tracking can enable real-time performance monitoring and benchmarking.

This study is subject to several limitations. First, the data were collected from a single tertiary referral hospital, which may limit the generalizability of the findings to other regions. Second, the data were collected over a single month (June 2017), which may not capture seasonal fluctuations in CS rates. This month’s selection was based on the availability of complete, standardized records and its alignment with institutional audit cycles, but it may introduce temporal bias. Third, the use of data from 2017 raises concerns about current relevance. However, recent reports and hospital-level trends suggest that cesarean section rates in Vietnam have remained consistently high over the past several years [7,28], and structural changes in maternal care policy have been limited [25]. Nonetheless, the age of the dataset is a notable limitation, and future research should include more recent and possibly longitudinal data to validate and update these findings.

## 5. Conclusions

This study provides one of the few institution-based analyses in Vietnam using the RTGCS to systematically evaluate cesarean section patterns. By identifying Group 5 and primary CSs in nulliparous women as key contributors, the findings offer evidence for targeted clinical interventions and support the adoption of VBAC and improved labor management protocols. While the data collected over a single month in 2017 may affect seasonal generalizability and reflect past institutional practices, the overall patterns remain highly relevant amid persistently elevated CS rates nationwide. Future research should explore provider behavior, patient preferences, and system-level factors driving unnecessary CS to inform sustainable national policy reforms. In addition to enhancing patient health literacy, hospital-based strategies are needed to address clinical drivers of high CS rates. These include expanding access to TOLAC with structured counseling and protocols, improving the selection criteria and timing for labor induction, increasing provider training in labor support techniques, and integrating real-time Robson-based audits into clinical governance. Such measures can support safer, more appropriate use of cesarean delivery within high-volume tertiary settings like Tu Du Hospital.

## Figures and Tables

**Table 1:** Cesarean section rate by Robson Ten-Group Classification System (N = 5287).

RTGCS Group	CS/Total Cases	Group Size (%)	CS Rate in Group (%)	Contribution to Overall CS Rate (%)
Group 1	289/1470	27.8%	19.7%	12.8%
Group 2	591/686	13.0%	86.2%	26.2%
Group 3	46/989	18.7%	4.7%	2.0%
Group 4	125/272	5.1%	46.0%	5.5%
Group 5	670/841	15.9%	79.7%	29.7%
Group 6	105/124	2.3%	84.7%	4.7%
Group 7	66/81	1.5%	81.5%	2.9%
Group 8	88/122	2.3%	72.1%	3.9%
Group 9	60/167	3.2%	35.9%	2.7%
Group 10	214/535	10.1%	40.0%	9.5%

RTGCS, Robson Ten-Group Classification System; CS, Cesarean section.

## Data Availability

Data is unavailable due to privacy restrictions.

## Abbreviations

The following abbreviations are used in this manuscript:

CS	Cesarean Section
EMR	Electronic Medical Record
RTGCS	Robson Ten-Group Classification System
TOLAC	Trial of Labor After Cesarean
VBAC	Vaginal Birth After Cesarean
WHO	World Health Organization
